# Characterization of the complete chloroplast genome of *Brassica oleracea* var. *italica* and phylogenetic relationships in Brassicaceae

**DOI:** 10.1371/journal.pone.0263310

**Published:** 2022-02-24

**Authors:** Zhenchao Zhang, Meiqi Tao, Xi Shan, Yongfei Pan, Chunqing Sun, Lixiao Song, Xuli Pei, Zange Jing, Zhongliang Dai

**Affiliations:** 1 Department of Vegetables and Flowers, Zhenjiang Institute of Agricultural Sciences, Jurong, China; 2 Department of Vegetables, Jiangsu Academy of Agricultural Sciences, Nanjing, China; 3 College of Agriculture and Life Science, Kunming University, Kunming, China; Huazhong University of Science and Technology, CHINA

## Abstract

Broccoli (*Brassica oleracea* var. *italica*) is an important *B*. *oleracea* cultivar, with high economic and agronomic value. However, comparative genome analyses are still needed to clarify variation among cultivars and phylogenetic relationships within the family Brassicaceae. Herein, the complete chloroplast (cp) genome of broccoli was generated by Illumina sequencing platform to provide basic information for genetic studies and to establish phylogenetic relationships within Brassicaceae. The whole genome was 153,364 bp, including two inverted repeat (IR) regions of 26,197 bp each, separated by a small single copy (SSC) region of 17,834 bp and a large single copy (LSC) region of 83,136 bp. The total GC content of the entire chloroplast genome accounts for 36%, while the GC content in each region of SSC,LSC, and IR accounts for 29.1%, 34.15% and 42.35%, respectively. The genome harbored 133 genes, including 88 protein-coding genes, 37 tRNAs, and 8 rRNAs, with 17 duplicates in IRs. The most abundant amino acid was leucine and the least abundant was cysteine. Codon usage analyses revealed a bias for A/T-ending codons. A total of 35 repeat sequences and 92 simple sequence repeats were detected, and the SC-IR boundary regions were variable between the seven cp genomes. A phylogenetic analysis suggested that broccoli is closely related to *Brassica oleracea* var. *italica* MH388764.1, *Brassica oleracea* var. *italica* MH388765.1, and *Brassica oleracea* NC_0441167.1. Our results are expected to be useful for further species identification, population genetics analyses, and biological research on broccoli.

## Introduction

Broccoli is a vegetable with a high nutrient content in *Brassica oleracea*. It possesses of wide range of nutrients, including vitamins A and K, antioxidants, β-carotene, calcium, riboflavin, and iron [1], as well as phytochemicals, such as phenols, flavonoids, glucosinolates, minerals, and selenium. The consumption of broccoli is beneficial to human health [2], exerting anti-inflammatory, anti-obesity, cholesterol-lowering, and anti-carcinogenic effects as well as high antioxidant activity [3, 4]. Broccoli was introduced to China as a special vegetable and was initially cultivated on a small scale. Over the past few decades, broccoli has played in increasing role in the booming vegetable industry and has become an increasing source of income for farmers.

Chloroplasts (cp) are crucial organelles in plant cells as a metabolic center of cellular reactions [5]. They play critical roles in carbohydrate metabolism, photosynthesis, and various molecular processes as well as in the regulation of physiology, growth, development, and stress responses [6, 7]. The typical cp genome of angiosperms has a quadripartite structure with a small single-copy (SSC) region and large single-copy region (LSC) region divided by two inverted repeat (IR) regions [8]. The gene content and organization of cp genomes are highly conserved; however, IR expansions and contractions, gene loss, inversions, and rearrangements have been reported [9]. Owing to their high conservation and slow rates of evolution, cp genomes are invaluable for phylogenetic classification, DNA barcoding, and genetic engineering [10, 11].

Broccoli crops from the *Brassica oleracea* group likely originated in the Mediterranean basin and are linked to closely related species, e.g., *Brassica cretica* and *Brassica montana* [12]. The selection and domestication processes led to the spread and exchange of genetic materials with other *Brassica oleracea* cultivars. Intense trade and human migration among several continents promoted the spread of the crop worldwide since the 15^th^ century, resulting in the development of new cultivars and hybrids, mainly in European and Asian countries. Adaptation to different soil and climatic conditions resulted from the cultivation and selection of genotypes with beneficial agronomical and qualitative traits [13].

Advances in high-throughput Illumina genome sequencing technologies have provided an opportunity to obtain and analyze the complete chloroplast genome of broccoli for analyses of its molecular and genomic characteristics. A sufficient knowledge of its genetic diversity is essential for the development of efficient strategies for its exploitation. Several complete cp genomes of *Brassica oleracea* are available in GenBank (Accession numbers KX681657.1, MH388765.1, MH388764.1, KX681654.1, KR233156.1, MG717288.1, MG717287.1, KX681655.1, and KX681656.1).

In this study, we sequenced and assembled the complete cp genome of broccoli cultivar 2001B (*B*. *oleracea* var. *italica*) for analyses of phylogenetic relationships with *B*. *oleracea* cultivars and other members of Brassicaceae. In particular, we *de novo* sequenced and assembled the complete cp genome of broccoli using the Illumina HiSeq2500 platform, followed by gene annotation and structural analyses, the identification of simple sequence repeat (SSR) markers, and reconstruction of evolutionary relationships among species in Brassicaceae. These results will hopefully improve our understanding of the cp genome and provide a theoretical basis for future scientific research on broccoli.

## Materials and methods

### DNA extraction and sequencing

Broccoli was planted in the experimental field of Zhenjiang Institute of Agricultural Sciences in Jurong, Jiangsu Province, China (N31°58’, E119°9’). Fresh leaves were collected and wrapped with tin foil, frozen with liquid nitrogen, and immediately stored at -80°C. Total genomic DNA was extracted from approximately 5 g of leaves with Plant DNA Isolation Reagent (Takara, USA) following the manufacturer’s protocol. An Agilent 2100 Bioanalyzer (Agilent Technologies, Santa Clara, CA, USA) and NanoDrop 2000 Microspectrophotometer were used to evaluate the quality and integrity of the extracted DNA. After purification, the DNA was employed to build a sequencing library according to the manufacturer’s instructions. The Illumina HiSeq2500 platform (San Diego, CA, USA) was utilized to construct paired-end (PE) libraries with insert sizes of 150 bp and sequenced according to standard protocols, including sample quality testing, library construction and quality testing, and library sequencing.

### Cp genome assembly and annotation

High-quality clean reads were generated by trimming and filtering the low-quality reads and sequencing adapters using Trimmomatic v. 0.3649. The clean reads were mapped onto the available cp genome reference of *B*. *oleracea* var. *capitata* (NCBI accession: KX681654.1) using Bowtie2(version 2. 2. 5) [14] with default parameters and preset options. All cp-like reads were assembled into contigs using SPAdes3.10.1 [15]. Then, the contigs were aligned again on the *Brasscia oleracea* var. *capitata* reference using the BLAST algorithm. The generated contigs and mate-pair reads were used for scaffolding using SSPACE(version 3.0) [16] to form a circular genome.

The tRNAs, rRNAs, and protein-coding genes of the plastome were annotated using the CpGAVAS online (http://www.herbalgenomics.org/0506/cpgavas) [17] and then manually corrected. BLAST(v2.2.31) and DOGMA (http://dogma.ccbb.utexas.edu/) were used to check the annotation results [18]. The online tool tRNAscan-SE with default settings (http://lowelab.ucsc.edu/tRNAscan-SE/) was applied to analyze the tRNAs [19]. The physical circular cp genome map was generated using OrganellarGenomeDRAW (http://ogdraw.mpimp-golm.mpg.de/index.shtml) [20] with default settings and checked manually. Relative synonymous codon usage (RSCU) was evaluated using CodonW v1.4.4 [21]. Long repetitive sequences and SSRs were analyzed using REPuter (http://bibiserv.techfak.uni-bielefeld.de/reputer/) [22] and MISA v1.0 (http://pgrc.ipk-gatersleben.de/misa/misa.html) [23] with the parameters are 1–8 (single base repeats 8 times and more), 2–5 (double base repeats 5 times and more), 3–3 (tribasic repeats 3 times and more), 4–3 (tetrabase repeats 3 times and more), 5–3 (pentabase repeats 3 times and more), respectively.

Sequencing data and gene annotations of *B*. *oleracea var*. *italica* were submitted to NCBI GenBank database (Accession Number: MN649876.1).

### Cp genome comparison

The newly generated genome (MN649876.1) was compared with the genomes of *Brassica oleracea* var. *italica* (Accession Number: MH388765.1), *Brassica oleracea* var. *capitata* (Accession Number: MG717287.1), *Brassica oleracea* var. *botrytis* (Accession Number: KX681655.1), and *Brassica oleracea* var. *gongylodes* (Accession Number: KX681656.1). And compare the boundaries between the LSC, IR and SSC regions in the chloroplast genome with other six genomes(*Arabidopsis thaliana*, *Capsella burse-pastoris*, *Brassica napus*, *Brassica juncea*, *Brassica nigra*, and *Bunias orientalis*) using mVISTA (http://genome.lbl.gov/vista/index.shtml) in the shuffle-LAGAN mode [24], with the annotation of *B*. *oleracea* var. *capitata* as a reference. The IRB-LSC, IRB-SSC, IRA-SSC, and IRA-LSC boundaries were compared among the seven species with the annotations of cp genomes available in GenBank.

### Phylogenetic analysis

The phylogenetic trees were constructed by aligning total chloroplast protein-coding sequences from 31 species in Brassicaceae obtained from the GenBank database, using *Carica papaya* (NC_010323.1) as an outgroup. MAFFTA version 7.017 [25] was used generate sequence alignments. FastTree v. 2.1.10 [26] was employed to construct a phylogenetic tree by the maximum likelihood (ML) method with the GTRGAMMA model and 1000 bootstrap replicates to evaluate node support.

## Results

### Characteristics of the broccoli cp genome

**Fig 1:**
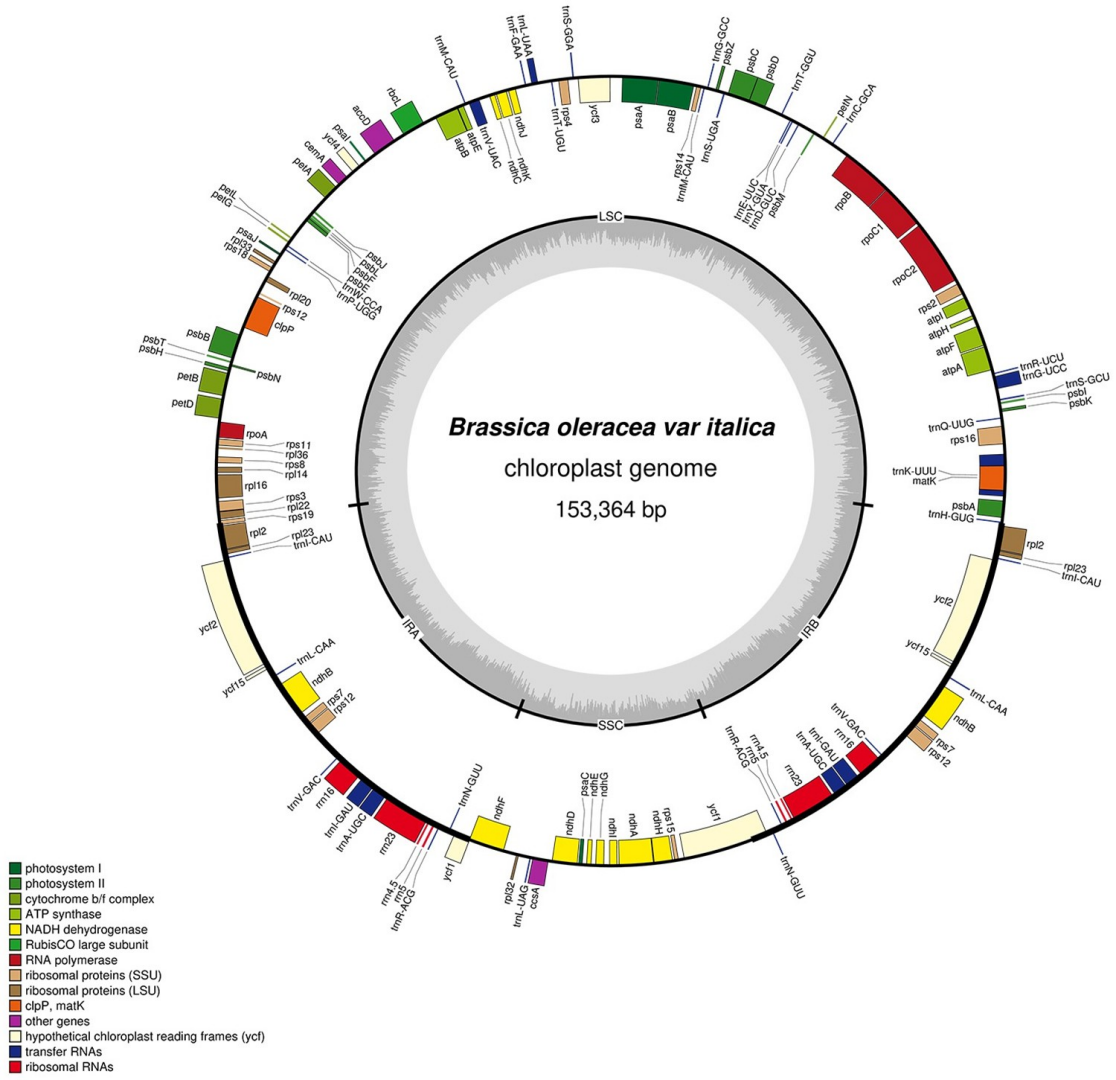
Physical map of the *B*. *oleracea* var. *italica* cp genome.

The newly generated genome (MN649876.1) was a typical quadripartite circular molecule 153,364 bp in length, containing a pair of two IR (IRA and IRB) regions of 26,197 bp each, separated by a SSC region of 17,834 bp and a LSC region of 83,136 bp (Fig 1 and Table 1). The AT and GC contents of overall cp genome were 63.64% and 36.36%, respectively. The cp genome had a biased base composition (31.36% A, 32.28% T, 17.86% G, and 18.5% C) with an overall GC content of 36.36%. The GC contents of the IR, LSC, and SSC regions were 42.35%, 34.15%, and 29.1%, respectively.

**Table 1:** Summary of cp genome of *B*. *oleracea* var. *italica*.

Features	Numerical value	Features	Numerical value
Genome size (bp)	153,364	GC content in SSC region (%)	29.1
LSC length (bp)	83,136	Gene number	133
SSC length (bp)	17,834	Protein-coding gene number	88
IR length (bp)	26,197	tRNA gene number	37
AT content (%)	63.64	rRNA gene number	8
GC content (%)	36.36	Gene number in LSC regions	85
GC content in IR region (%)	42.35	Gene number in SSC regions	14
GC content in LSC region (%)	34.15	Gene number in IR regions	34

The genome harbored 133 genes, including 88 protein-coding genes (PCGs) (79 PCG species), 37 tRNA genes (30 tRNA species), and 8 rRNA genes (4 rRNA species) (Fig 1, Tables 1 and 2). Among these, 15 genes encoded a small ribosomal subunit (SSU), 11 encoded a large ribosomal subunit (LSU), and 4 genes encoded the DNA-directed RNA polymerase. Forty-five genes were associated with photosynthesis, including 5 encoding photosystem I and 15 encoding the photosystem II complex, 12 for subunits of NADH dehydrogenase, 6 for the cytochrome *b/f* complex, 6 for different subunits of ATP synthase, and one for the Large subunit of rubisco. Five genes were associated with functions other than self-replication and photosynthesis, and eight genes had unknown functions. Thirty-four genes, including 14 tRNA genes, 2 *rps7*, 2 *ndhB*, 2 *rpl2*, 2 *rpl23*, 2 *rrn5*, 2 *rrn4*.*5*, 2 *rrn23*, 2 *rrn16*, 2 *ycf2*, and 2 *ycf15* were duplicated in the IR regions. Most of the genes occurred as a single copy, and 18 gene species occurred in two copies, including 4 rRNA species (rrn4.5, rrn5, rrn16, and rrn23), 7 tRNA species (*trnA-UGC*, *trnI-GAU*, *trnN-GUU*, *trnV-GAC*, *trnL-CAA*, *trnI-CAU*, and *trnR-ACG*), and 7 PCG species (*rps7*, *rpl2*, *rpl23*, *ndhB*, *ycf1*, *ycf2*, and *ycf15*), in addition, one PCG species (*rps12*) occurred in three copies. Except for *ycf1* and *rps12* residing within the LSC region, all other 15 duplicated gene species were completely located within the IR regions. Nine PCG species (*rps16*, *rpl2*, *rpl16*, *rpoC1*, *ndhA*, *ndhB*, *petB*, *petD*, and *atpF*) and five tRNA species (*trnA-UGC*, *trnI-GAU*, *trnK-UUU*, *trnL-UAA*, and *trnV-UAC)* contained a single intron, while three other PCG species (*rps12*, *ycf3*, and *clpP)* harbored two introns (Tables 2 and 3). The *trnK-UUU* gene had the largest intron (2557 bp), followed by the *ndhA* gene (1098 bp), whereas *trnL-UAA* has the smallest intron (311 bp). The intron in the *trnK-UUU* gene was 2555 bp, and the *matK* gene was contained within the intron.

**Table 2:** Gene contents in the cp genome of *B*. *oleracea* var. *italica*.

Category	Group of genes	Gene names	Account
**Self-replication**	Ribosomal RNA genes	*rrn4*.*5*^a^ (×2), *rrn5*^a^ (×2), *rrn16*^a^(×2), *rrn23*^a^ (×2)	8
	Transfer RNA genes	*trnA-UGC*^a^^,^*(×*2)*, *trnC-GCA*, *trnD-GUC*, *trnE-UUC*, *trnF-GAA*, *trnG-GCC*, trnG-UCC*, *trnH-GUG*, *trnI-CAU*^a^*(*×*2)*, *trnI-GAU*^a^^,^*(×*2)*, trnK-UUU*, *trnL-CAA*^a^*(*×*2)*, trnL-UAA*, *trnL-UAG*, *trnM-CAU*, *trnN-GUU*^a^*(*×*2)*, *trnP-UGG*, *trnQ-UUG*, *trnR-ACG*^a^*(*×*2)*, *trnR-UCU*, *trnS-GCU*, *trnS-GGA*, *trnS-UGA*, *trnT-GGU*, *trnT-UGU*, *trnV-GAC*^a^*(*×*2)*, trnV-UAC*, *trnW-CCA*, *trnY-GUA*, *trnfM-CAU*	37
	Small subunit ribosomal proteins (SSU)	*rps2*, *rps3*, *rps4*, *rps7*^a^ (×2), *rps8*, *rps11*, *rps12*^b^^,^** (×3), *rps14*, *rps15*, rps16*, *rps18*, *rps19*	15
	Large subunit ribosomal proteins (LSU)	*rpl2*^a^^,^* (×2), *rpl14*, rpl16*, *rpl20*, *rpl22*, *rpl23*^a^ (×2), *rpl32*, *rpl33*, *rpl36*	11
	RNA polymerase	*rpoA*, *rpoB*, rpoC1*, *rpoC2*	4
**Photosynthesis**	Photosystem I	*psaA*, *psaB*, *psaC*, *psaI*, *psaJ*	5
	Photosystem II	*psbA*, *psbB*, *psbC*, *psbD*, *psbE*, *psbF*, *psbH*, *psbI*, *psbJ*, *psbK*, *psbL*, *psbM*, *psbN*, *psbT*, *psbZ*	15
	NADH dehydrogenase	ndhA*, *ndhB*^a^^,^* (×2), *ndhC*, *ndhD*, *ndhE*, *ndhF*, *ndhG*, *ndhH*, *ndhI*, *ndhJ*, *ndhK*	12
	Cytochrome b/f complex	*petA*, petB*, petD*, *petG*, *petL*, *petN*	6
	ATP synthase	*atpA*, *atpB*, *atpE*, atpF*, *atpH*, *atpI*	6
	Large subunit of rubisco	*rbcL*	1
**Other genes**	Subunit of acetyl-CoA	*accD*	1
	Envelope membrane protein	*cemA*	1
	Maturase	*matK*	1
	Protease	*clpP* **	1
	C-type cytochrome synthesis gene	*ccsA*	1
**Genes of unknown function**	Conserved hypothetical chloroplast ORF	*ycf1*^a^ (×2), *ycf2* ^a^ (×2), *ycf15* ^a^ (×2), ycf3**, *ycf4*	8

Note:

^a, b^The letters indicate the gene wite two copes and three copes, respectively.

*, ** The symbols indicate the gene with one intron and two introns, respectively.

**Table 3:** Lengths of introns and exons in genes in the *B*. *oleracea* var. *italica* cp genome.

Gene	Location	Exon I (bp)	Intron I (bp)	Exon II (bp)	Intron II (bp)	Exon III (bp)
*trnK-UUU*	LSC	37	2557	35		
*rps16*	LSC	46	859	221		
*trnG-UCC*	LSC	18	716	54		
*atpF*	LSC	145	721	410		
*rpoC1*	LSC	432	778	1611		
*ycf3*	LSC	126	782	228	732	153
*trnL-UAA*	LSC	37	311	50		
*trnV-UAC*	LSC	39	601	35		
*clpP*	LSC	68	570	295	938	228
*petB*	LSC	6	784	639		
*petD*	LSC	8	733	475		
*rpl16*	LSC	9	1058	405		
*rpl2*	IRB	393	684	435		
*ndhB*	IRB	777	679	762		
*rps12*	IRB	231	-	27	539	102
*trnI-GAU*	IRB	37	809	35		
*trnA-UGC*	IRB	38	800	35		
*ndhA*	SSC	552	1098	531		
*trnA-UGC*	IRA	38	800	35		
*trnI-GAU*	IRA	37	809	35		
*ndhB*	IRA	777	679	762		
*rpl2*	IRA	393	684	435		
*rps12*	IRA	102	-	231	539	27

For comparative analyses, information from the new genome and other genomes from the GenBank was compared (Tables 4 and 5). Except for the genome size of *B*. *oleracea var*. *Italica* (MH388765.1) which is 153365bp, the other genome sizes are 1553364bp (Table 5). The tRNA genes were exactly the same detected in MN649876.1 and MH388765.1. Besides, a pseudogene, *rps19*, in the MH388765.1 cp genome was not detected in other species. KX681655.1 and KX681656.1 lost 11 genes detected in MN649876.1 (Table 4). In addition, Compared to the reference genome, *Brasscia gongylodes* (KX681656.1) genome contains two indels and nine SNPs, one of the indels involves the *rpoC2* gene, *Brasscia italica* (MH388765.1) genome contains one indel which involves the *ycf1* gene and five SNPs, *Brasscia capitata* (MG717287.1)) includes 12 SNPs, 10 of which involves the *ycf1* gene, and *Brasscia botrytis* (KX681655.1) includes 2 SNPs (Table 5).

**Table 4:** Differences in annotated genes between the newly generated genome (MN649876.1) and other *Brassica oleracea* genomes.

Position	*Brasscia italica* MN649876.1	*Brasscia italica* MH388765.1	*Brasscia capitata* MG717287.1	*Brasscia botrytis* KX681655.1	*Brasscia gongylodes* KX681656.1
1656..1692,4248..4285	*trnK-UUU*	*trnK-UUU*	*trnK-UUU*	-	-
8335..8368,9081..9123	-	-	*trnG*	-	-
35478..35549	*trnG-UCC*	*trnG-UCC*	*trnG*	-	-
46097..46131,46445..46494	*trnL-UAA*	*trnL-UAA*	*trnL*	-	-
49885..49920,50520..50559	*trnV-UAC*	*trnV-UAC*	*trnV*	-	-
85288..85362	*trnI-CAU*	*trnI-CAU*	*trnI*	-	-
93224..93306	*trnL-CAA*	*trnL-CAA*	*trnL*	-	-
113194..113275	*trnL-UAG*	*trnL-UAG*	*trnL*	-	-
133052..133088,133887..133924	*trnA-UGC*	*trnA-UGC*	*trnA*	-	-
133989..134029,134837..134869	*trnI-GAU*	*trnI-GAU*	*trnI-GAU*	-	-
136816..136887	*trnV-GAC*	*trnV-GAC*	*trnV-GAC*	-	-
143195..143277	*trnL-CAA*	*trnL-CAA*	*trnL-CAA*	-	-
153253..153363	-	*rps19 (pseudo)*	-	-	-

**Table 5:** Differences in genome size and genome divergence (SNPs and Indels) between the newly generated genome (MN649876.1) and other *Brassica oleracea* genomes.

Sort		Position		*Brasscia italica* MN649876.1 (Ref)	*Brasscia italica* MH388765.1(Alt)	*Brasscia capitata* MG717287.1(Alt)	*Brasscia botrytis* KX681655.1(Alt)	*Brasscia gongylodes* KX681656.1(Alt)	Gene
		Start	End						
genome size(bp)		-	-	153364	153365	153364	153364	153364	-
Indel	1	15623	15624	GT	-	-	-	-	*rpoC2*
		15623	15623	-	-	-	-	G	
	2	26564	26564	A	-	-	-	-	-
		26563	26564	-	-	-	-	AT	
	3	124803	124803	T	-	-	-	-	*ycf1*
		124803	124804	-	TT	-	-	-	
SNP	1	70595	70595	A	-	-	T	-	-
	2	79477	79477	T	-	-	A	-	-
	3	264	264	A	-	-	-	T	-
	4	265	265	A	-	-	-	G	-
	5	266	266	C	-	-	-	T	-
	6	267	267	A	-	-	-	T	-
	7	3778	3778	T	-	-	-	A	-
	8	7351	7351	T	-	-	-	A	-
	9	70595	70595	A	-	-	-	T	-
	10	75696	75696	C	-	-	-	G	-
	11	79477	79477	T	-	-	-	A	-
	12	70595	70595	A	-	T	-	-	-
	13	79477	79477	T	-	A	-	-	-
	14	124794	124794	T	-	A	-	-	*ycf1*
	15	124802	124802	A	-	T	-	-	*ycf1*
	16	124970	124970	A	-	G	-	-	*ycf1*
	17	124971	124971	G	-	A	-	-	*ycf1*
	18	124972	124972	A	-	T	-	-	*ycf1*
	19	124977	124977	C	-	T	-	-	*ycf1*
	20	124979	124979	A	-	G	-	-	*ycf1*
	21	124985	124985	T	-	A	-	-	*ycf1*
	22	124986	124986	C	-	T	-	-	*ycf1*
	23	124987	124987	G	-	T	-	-	*ycf1*
	24	36	36	A	G	-	-	-	-
	25	56	56	A	G	-	-	-	-
	26	7351	7351	T	A	-	-	-	-
	27	70595	70595	A	T	-	-	-	-
	28	79477	79477	T	A	-	-	-	-

Note: Ref, Alt represent reference and alter, respectively.

### Examination of codon usage frequency

**Fig 2:**
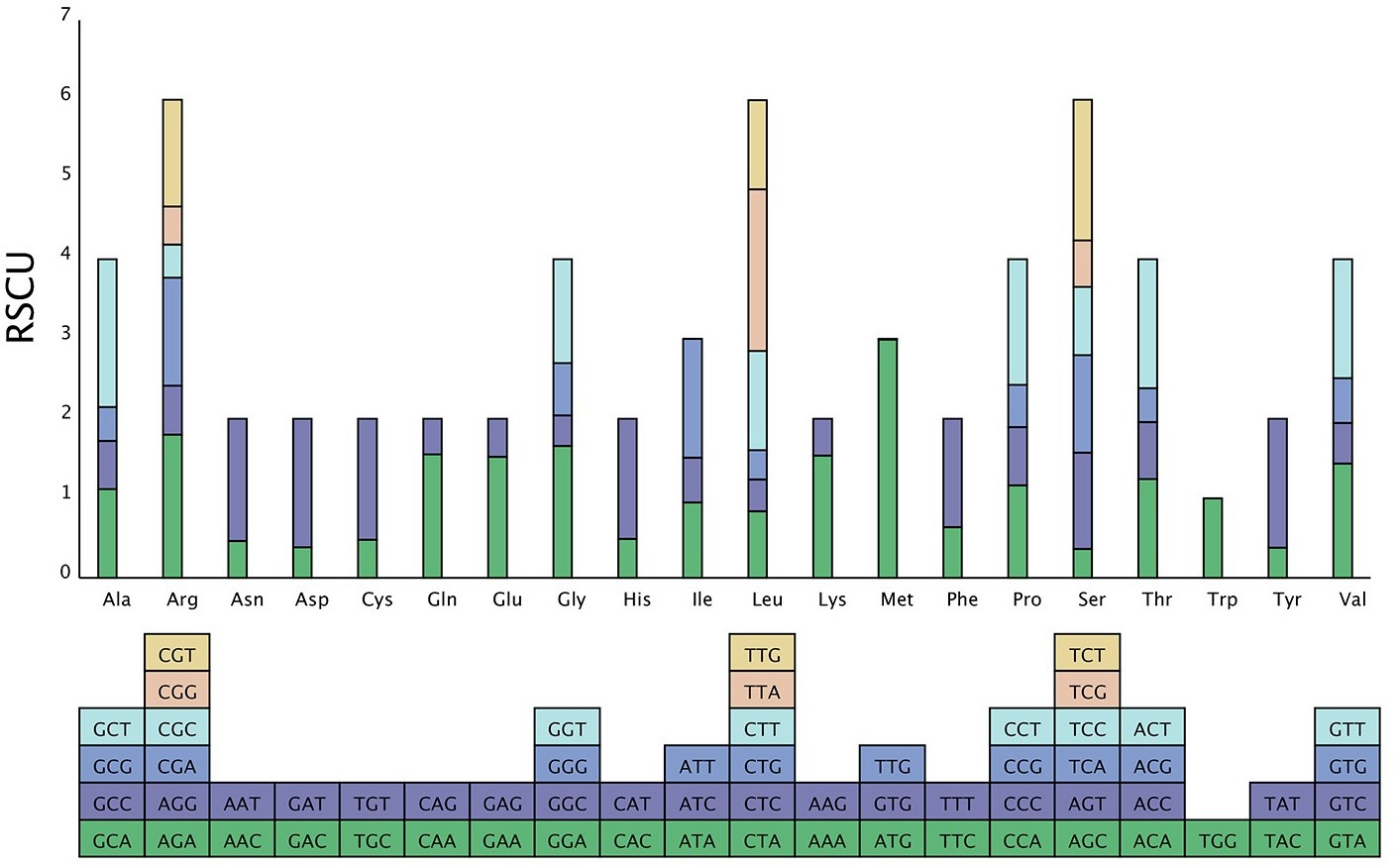
Codon contents of 20 amino acid and stop codons in all protein-coding genes of the broccoli cp genome.

According to the coding sequence (CDS), the relative synonymous codon usage frequency (RSCU) and codon usage frequency were estimated (Table 6, Fig 2). All protein-coding genes in the cp genome were composed of 26,681 codons. Among these codons, the termination codons were UAA, UAG, and UGA. AUG encoding methionine had the highest RSCU value (2.9901). The most abundant amino acid in the protein-coding genes was leucine (2829 codons, approximately 10.6% of the total), compared with only 325 codons (1.22%) for cysteine.

**Table 6:** Codon usage in the *B*. *oleracea* var. *italica* cp genome.

Amino Acid	Codon	Number	RSCU	tRNA	Amino Acid	Codon	Number	RSCU	tRNA
Ter	UAA	52	1.7931		Met	GUG	1	0.0051	*trnM-CAU*
Ter	UAG	22	0.7587		Met	UUG	1	0.0051	
Ter	UGA	13	0.4482		Asn	AAC	304	0.4628	*trnN-GUU*
Ala	GCA	382	1.116	*trnA-UGC*	Asn	AAU	1010	1.5372	
Ala	GCC	206	0.602		Pro	CCA	310	1.1632	*trnP-UGG*
Ala	GCG	146	0.4264		Pro	CCC	195	0.7316	
Ala	GCU	635	1.8552		Pro	CCG	141	0.5292	
Cys	UGC	80	0.48	*trnC-GCA*	Pro	CCU	420	1.576	
Cys	UGU	247	1.52		Gln	CAA	740	1.5514	*trnQ-UUG*
Asp	GAC	202	0.3854	*trnD-GUC*	Gln	CAG	214	0.4486	
Asp	GAU	846	1.6146		Arg	AGA	472	1.7982	*trnR-UCU*
Glu	GAA	1061	1.5212	*trnE-UUC*	Arg	AGG	161	0.6132	
Glu	GAG	334	0.4788		Arg	CGA	357	1.3602	
Phe	UUC	518	0.64	*trnF-GAA*	Arg	CGC	109	0.4152	
Phe	UUU	1101	1.36		Arg	CGG	125	0.4764	
Gly	GGA	733	1.6592	*TrnG-UCC*	Arg	CGU	351	1.3374	*trnR-ACG*
Gly	GGC	168	0.3804	*trnG-GCC*	Ser	AGC	125	0.3654	*trnS-GCU*
Gly	GGG	291	0.6588		Ser	AGU	413	1.2066	
Gly	GGU	575	1.3016		Ser	UCA	420	1.227	*trnS-UGA*
His	CAC	149	0.491	*trnH-GUG*	Ser	UCC	293	0.8556	*trnS-GGA*
His	CAU	458	1.509		Ser	UCG	199	0.5814	
Ile	AUA	726	0.9471	*TrnI-CAU*	Ser	UCU	604	1.7646	
Ile	AUC	432	0.5634	*trnI-GAU*	Thr	ACA	429	1.2424	*trnT-UGU*
Ile	AUU	1142	1.4895		Thr	ACC	247	0.7156	*trnT-GGU*
Lys	AAA	1167	1.5356	*trnK-UUU*	Thr	ACG	147	0.4256	
Lys	AAG	353	0.4644		Thr	ACU	558	1.6164	
Leu	CUA	395	0.8376	*trnL-UAG*	Val	GUA	512	1.434	*trnV-UAC*
Leu	CUC	189	0.4008		Val	GUC	182	0.51	*trnV-GAC*
Leu	CUG	173	0.3672		Val	GUG	201	0.5632	
Leu	CUU	587	1.245		Val	GUU	533	1.4928	
Leu	UUA	955	2.0256	*trnL-UAA*	Trp	UGG	452	1	*trnW-CCA*
Leu	UUG	530	1.1238	*trnL-CAA*	Tyr	UAC	188	0.381	*trnY-GUA*
Met	AUG	602	2.9901	*trnfM-CAU*	Tyr	UAU	799	1.619	

The codon-anticodon recognition patterns of the cp genome showed that 30 tRNAs contained codons corresponding to 20 essential amino acids for protein biosynthesis. The AT contents at the first, second, and third codon positions were 55.3%, 62.53%, and 71.21%, respectively. Moreover, of all 66 codons, the RSCU values for 31 codons were >1, and most (13/16, 93.5%) ended with base A or U, whereas 34 codons had RSCU values of <1, and most of these (16/15, 91.2%) ended with base C or G. *Trp* was encoded by only a UGG codon, indicating no biased usage (RSCU = 1).

### Analyses of repeat sequences and SSRs

A total of 35 repeat sequences, including 12 forward (F), 20 palindromic (P), and 3 reverse (R) repeats were detected using REPuter in the broccoli cp genome (Table 7). Repeat lengths were generally between 30 to 47 bp. LSC, SSC, and IR regions harbored 22, 7, and 12 repeats, respectively. Most repeats were mainly located in intergenic spaces (IGS), *ycf*, and intron sequences, whereas 13 repeats were located in *psaA*, *psaB*, *trnS-GCU*, *trnS-GGA*, and *trnS-UGA*.

**Table 7:** Repeat sequences in the broccoli chloroplast genome.

ID	Repeat Start	Type	Size(bp)	Repeat Start2	Mismatch(bp)	E-Value	Gene	Region
1	61539	F	47	61583	0	3.34E-19	IGS	LSC;LSC
2	37725	F	46	39949	-3	5.48E-13	psaB;psaA	LSC;LSC
3	75674	P	45	75674	-1	7.21E-16	petD;petD	LSC;LSC
4	37704	F	43	39928	-3	2.85E-11	psaB;psaA	LSC;LSC
5	28145	P	40	28145	0	5.47E-15	IGS	LSC;LSC
6	73171	P	40	73175	-3	1.46E-09	IGS	LSC;LSC
7	97778	F	37	119318	-3	7.35E-08	IGS;ndhA	IRb;SSC
8	119318	P	37	138687	-3	7.35E-08	ndhA;IGS	SSC;IRa
9	9182	P	36	9182	0	1.40E-12	IGS	LSC;LSC
10	172	P	36	172	-2	7.94E-09	IGS	LSC;LSC
11	106664	F	34	106696	-2	1.13E-07	IGS	IRb;IRb
12	106664	P	34	129772	-2	1.13E-07	IGS	IRb;IRa
13	106696	P	34	129804	-2	1.13E-07	IGS	IRb;IRa
14	129772	F	34	129804	-2	1.13E-07	IGS	IRa;IRa
15	6223	P	32	6223	0	3.59E-10	IGS	LSC;LSC
16	88070	F	32	88091	-3	4.80E-05	ycf2;ycf2	IRb;IRb
17	88070	P	32	148379	-3	4.80E-05	ycf2;ycf2	IRb;IRa
18	88091	P	32	148400	-3	4.80E-05	ycf2;ycf2	IRb;IRa
19	148379	F	32	148400	-3	4.80E-05	ycf2;ycf2	IRa;IRa
20	7603	F	31	34397	-3	1.74E-04	trnS-GCU;trnS-UGA	LSC;LSC
21	61473	P	30	61473	0	5.74E-09	IGS	LSC;LSC
22	7604	P	30	43913	-1	5.16E-07	trnS-GCU;trnS-GGA	LSC;LSC
23	42810	F	30	97787	-2	2.25E-05	ycf3;IGS	LSC;IRb
24	42810	P	30	138685	-2	2.25E-05	ycf3;IGS	LSC;IRa
25	64605	P	30	64605	-2	2.25E-05	IGS	LSC;LSC
26	122596	P	30	123176	-2	2.25E-05	IGS;ycf1	SSC;SSC
27	124278	P	30	124278	-2	2.25E-05	ycf1;ycf1	SSC;SSC
28	3753	F	30	120284	-3	6.29E-04	trnK-UUU;ndhA	LSC;SSC
29	34398	P	30	43913	-3	6.29E-04	trnS-UGA;trnS-GGA	LSC;LSC
30	34466	P	30	43851	-3	6.29E-04	trnS-UGA;trnS-GGA	LSC;LSC
31	65897	P	30	65948	-3	6.29E-04	IGS	LSC;LSC
32	124225	F	30	124252	-3	6.29E-04	ycf1;ycf1	SSC;SSC
33	173	R	30	34492	-3	6.29E-04	IGS	LSC;LSC
34	185	R	30	112594	-3	6.29E-04	IGS	LSC;SSC
35	34491	R	30	174	-3	6.29E-04	trnS-UGA;IGS	LSC;LSC

Note: IRa and IRb,represent a pair of inverted repeats. SSC and LSC represent a small single copy region and a lager single copy region, respectively

**Fig 3:**
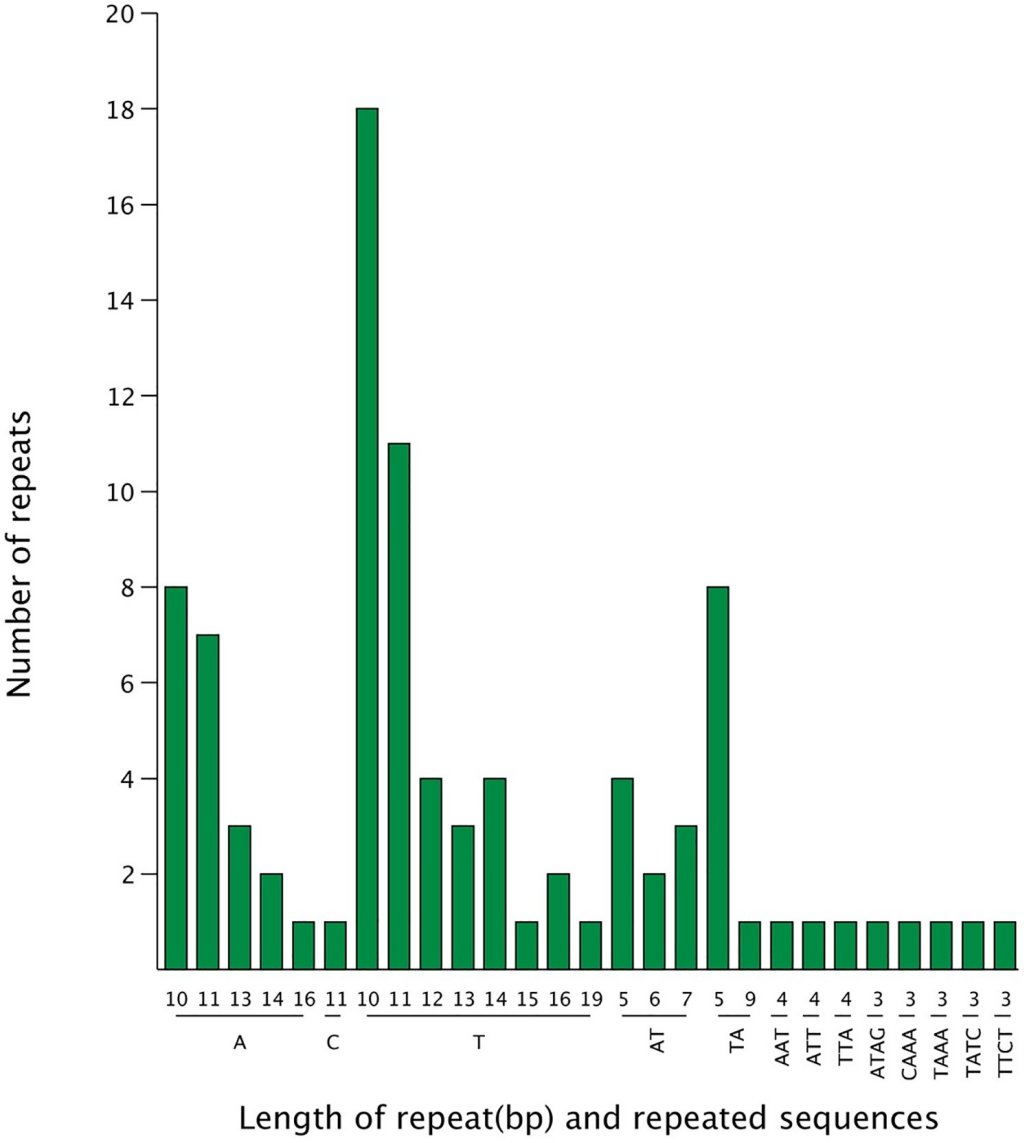
Statistical summary of repeat sequences in the cp genome of broccoli.

A total of 92 SSRs, including 66 mononucleotides (P1), 18 dinucleotides (P2), 3 trinucleotides (P3), and 5 tetranucleotides were explored. Most were distributed in the LSC (58, 63.00%) and SSC regions (22, 23.9%), with some in the IR region (12,13.00%) (Tables 8 and 9, Fig 3). One SSRs belonged to the C repeat units and the others belonged to the A and T types (98.49%), while dinucleotides included TA and AT repeats. Trinucleotides were the last prevalent with the lowest number of repeat units (3). Moreover, 37 repeats were found in different genes, and the remaining were found in intergenic regions.

**Table 8:** Number of SSRs distributed in the SSC, LSC, and IR regions.

Region	Exon	Intron	Intergenic	Number	Proportion
SSC	13	4	5	22	23.90%
LSC	6	12	40	58	63.00%
IR	2	0	10	12	13.00%

**Table 9:** Distribution of SSRs in the broccoli cp genome.

SSR type	Unit	Length	Number	Genomic position (gene)
P1	A	10	8	12446–12455, 12867–12876, 41568–41577, 50050–50059_(trnV-UAC), 66012–66021, 109314–139323_(ndhF), 122605–122614, 138192–138201
		11	7	26937–26947, 60220–60230, 64103–64113,82925–82935, 112599–112609, 119762–119772_(ndhA), 137413–137423
		13	3	67123–67135, 124958–124970_(ycf1), 126037–126049_(ycf1)
		14	2	113669–113682_(ccsA), 140343–140356
		16	1	30260–30275
	T	10	18	15624–15633_(rpoC2), 16763–16772_(rpoC2), 25247–25256_(rpoB), 28408–28417, 29383–29392, 48803–48812, 53171–53180, 55633–55642, 64126–64135,70288–70297_(clpP), 80677–80686_(rpl16), 81154–81163_(rpl16), 81550–81559_(rpl16), 81661–81670, 98300–98309, 123187–123196_(ycf1), 124444–124453_(ycf1), 127178–127187_(ycf1)
		11	11	3978–3988_(trnK-UUU), 6815–6825, 7777–7787, 12467–12477, 17512–17522_(rpoC2), 74110–74120_(petB), 99078–99088, 120304–120314_(ndhA), 120317–120327_(ndhA), 123208–123218_(ycf1), 126007–126017_(ycf1)
		12	4	4061–4072_(trnK-UUU), 63338–63349, 70265–70276_(clpP), 123096–123107_(ycf1)
		13	3	47324–47336, 77038–77050_(rpoA), 125310–125322_(ycf1)
		14	4	3769–3782_(trnK-UUU), 50869–50882, 96145–96158, 124990–125003_(ycf1)
		15	1	12160–12174_(atpF)
		16	2	7336–7351, 111781–111796
		19	1	124803–124821_(ycf1)
	C	11	1	62109–62119
P2	AT	10	4	7917–7926, 107448–107457, 129044–129053, 143015–143024
		12	2	13319–13330, 112614–112625
		14	3	3756–3769_(trnK-UUU), 30560–30573, 120287–120300_(ndhA)
	TA	10	8	4557–4566, 6234–6243, 7841–7850, 18884–18893_(rpoC2), 26480–26489, 61869–61878, 93476–93485, 122815–122824_(ycf1)
		18	1	111597–111614
P3	AAT	12	1	12612–12623
	TTA	12	1	26447–26458
	ATT	12	1	45612–45623
P4	CAAA	12	1	27991–28002
	TTCT	12	1	34268–34279
	TAAA	12	1	45436–45447
	TATC	12	1	47120–47131
	ATAG	12	1	111359–111370_(ndhF)

### IR junction characteristics

**Fig 4:**
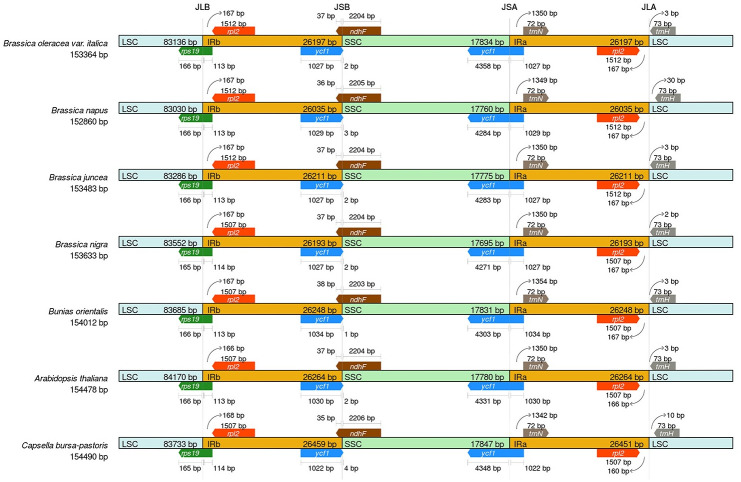
Comparison of boundaries between the LSC, IR, and SSC regions in chloroplast genomes of seven species. Genes are depicted by colored boxes. Boxes above or below the main line indicate adjacent border genes.

The expansion and contraction of IR-SSC and IR-LSC boundaries of seven species, including *B*. *oleracea* var. *italica*, *A*. *thaliana*, *C*. *bursa−pastoris*, *B*. *napus*, *B*. *juncea*, *B*. *nigra*, and *Bunias orientalis*, were compared (Fig 4). In the figure, JLB, JLA, JSB, JSA represent for IRb/LSC, IRa/LSC, IRb/SSC, and IRa/SSC junction, respectively. The IR sizes of the LSC, IR, and SSC regions were similar in the cp genomes of the seven species, and the IR length varied from 26,035 bp in *B*. *napus* to 26,459 bp in *C*. *bursa−pastoris* (accession number: AP009371). The JLB border was within the coding region of *rps19* in the above seven species and only 1 base par difference in location across different cp genomes. The two genes *ycf1* and *ndhF* crossed the JSB junction. Most of the *ycf1* gene in the seven species was located in the IRB region and 1–4 bp was located in the SSC region. Overlap between *ycf1* and *ndhF* was detected at the JSB boundary in all studied cp genomes, with lengths of 35 bp to 38 bp. The *ycf1* gene crossed the JSA region in all cp genomes, and its length reflected changes in the JSA region. The tRNA noncoding gene *trnH-GUG* in the seven species were all within the LSC region, located 2–30 bp from the JLA boundary. These results suggested that the IR border shifts were relatively minor, involving only a small number of genes, with differences in gene overlap lengths and the distance of *trnH-GUG* at the junction of JLA boundaries.

### Phylogenetic analysis

**Fig 5:**
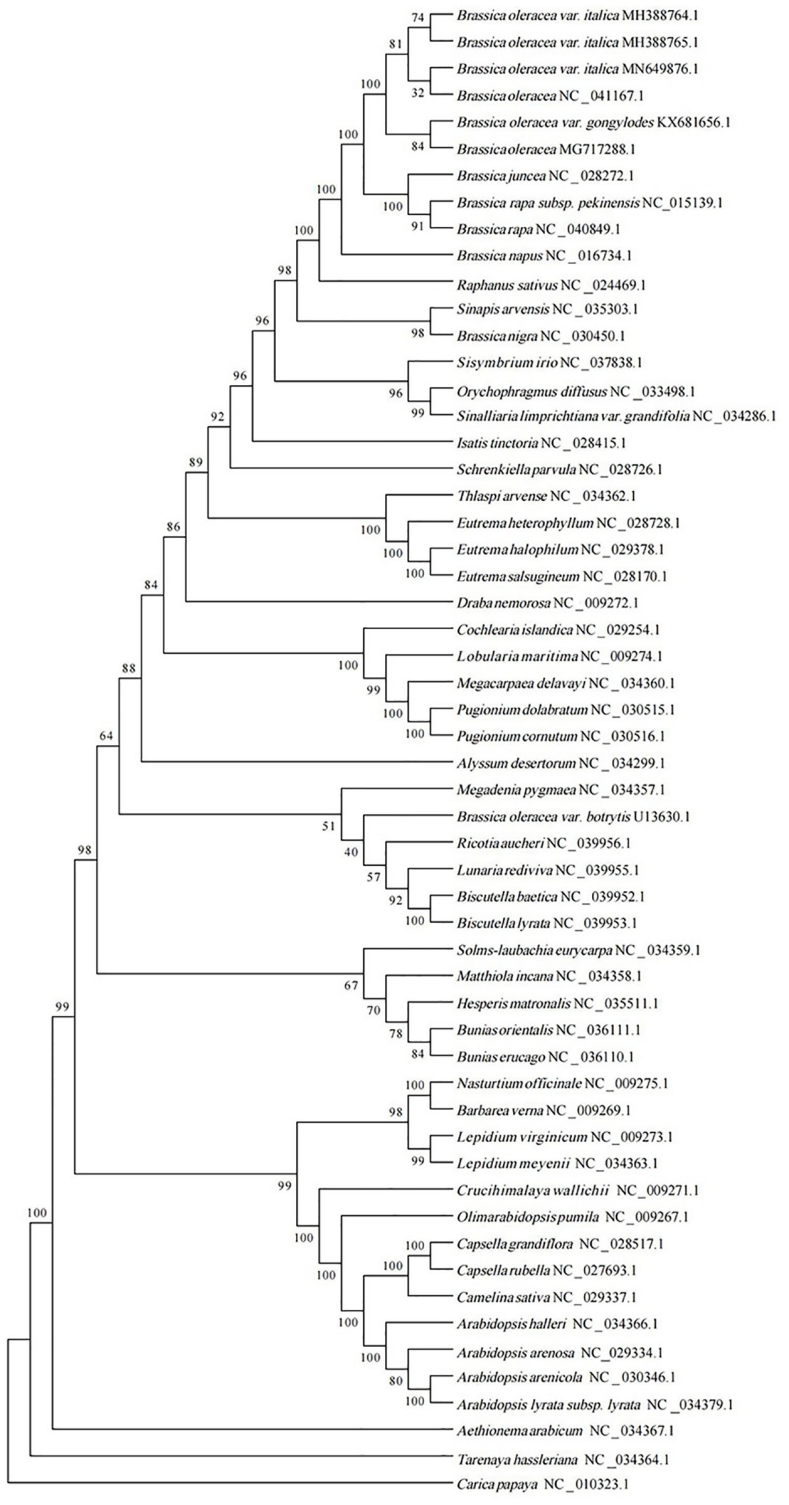
Phylogenetic tree inferred by the maximum likelihood method based on the complete cp genomes from 56 species. Bootstrap support values are shown at the nodes.

cpDNA-based phylogenetic analyses have provided insight into evolutionary relationships, population genetics, and classification in different plant taxa [27]. To investigate the taxonomic status and evolutionary relationships of *Brassica oleracea* var. *italica* within Brassicaceae, ML phylogenetical analyses were performed based on 56 complete cp genome sequences (Fig 5). The phylogenetic analysis revealed that all *B*. *oleracea* cultivars were closely related, forming a well-supported clade. The newly generated genome (Accession Number: MN649876.1) was classified as *B*. *oleracea* and formed a clade with *Brassica oleracea* (NC_041167.1). The two *Brassica oleracea* var. *italica* cultivars MH388765.1 and MH388764.1 formed a clade. *B*. *oleracea* var. *Gongylodes* and *B*. *oleracea* MG717288.1 formed a clade. The phylogenetic results clearly elucidate the position of *B*. *oleracea* var. *italica* within in Brassicaceae and provide a basis for future evolutionary studies.

## Discussion

*B*. *oleracea* var. *italica* is an important vegetable among *B*. *oleracea* cultivars. In general, the gene content and genome organization of land plant chloroplast genomes are more highly conserved than those of mitochondrial and nuclear genomes. However, gene losses and inversions had been reported in Asteraceae, Leguminosae, and Gentianaceae [28–30]. In the present study, we compared the complete cp genomes and gene annotations of various *B*. *oleracea* cultivars with data available in the GenBank database. The size of the cp genome obtained in this study was similar to those of other *B*. *oleracea* varieties. However, the number of annotated genes differed among genomes; this may be explained by incomplete data, gene losses, or interspecific differences.

Our results indicated that the DNA GC content was not evenly distributed among genomic regions. The GC content in the IR region was higher than those in other regions, possibly because the GC content (an indicator of species relationships) of the four rRNAs in this region was high [11, 31]. The newly sequenced broccoli genome contained 133 genes, with high conservation in composition and arrangement, including self-replication genes, photosynthetic genes, other functional genes, and genes with unknown functions, consistent with previous research [32]. Furthermore, 23 genes contained one intron or two introns, and *trnR-UKK* had the largest intron. Introns play crucial roles in the regulation of gene expression depending on conditions and on the location [33]. Coding usage is a key factor in cp genome evolution. In the broccoli cp genome, the most and least frequent amino acids were leucine and cysteine, respectively, as observed in other angiosperm genomes, such as *Ananas comosus*, *Decaisnea insignis*, *Nasturtium officinale*, and *Magnolia zenii* [34–36]. In the broccoli cp genome, AT was preferred over GC, especially at the second and third codon positions (62.53% and 71.21%, respectively), consistent with results obtained for many terrestrial species [37].

A repeat analysis revealed 12 forward, 20 palindromic, and 3 reverse repeats in the broccoli cp genome. Most of these repeats were located in intron sequences, intergenic spacers, and the *ycf* gene, but several occurred in CDS regions and tRNAs. Repeat sequences are involved in sequence variation, genome rearrangements, and many rearrangement endpoints in algal and angiosperm genomes [38, 39]. The organization of cp genome sequences is highly conserved and the SSR primer for cp genomes can be inherited across genera and species. Accordingly, SSRs are widely used as molecular markers for genetic linkage map construction, population genetic analyses, polymorphism identification, plant breeding, and taxonomic analyses [40]. A total of 92 SSRs were obtained in this study, and 66 (71.7%) SSRs belonged to the P1 type, among which 65 (70.7%) belonged to A and T repeat units, while TA and AT repeats belonged to the P2 type. These findings agree with previous results [41, 42]. The phylogenetic analysis yielded 53 notes with bootstrap values, among which 21 and 36 notes had bootstrap values greater than 100% and 90%, respectively. In this present study, the phylogenetic trees demonstrated that Brassica nigra and S. arvensis were clustered into one subgroup, which was consistent with others research [43]. And the newly generated genome (Accession Number: MN649876.1) was closely related to NC_041167.1.

Plant cp genomes are considered highly conserved; however, the sizes and LSC/IRb/SSC/IRa boundaries change due to contraction or expansion at the borders of the IR region [44]. Our results indicated that divergence in the IR border between seven species was related to the different positions of four genes, *rps19*, *ycf1*, *ndhF*, and *trnH-GUG*, in agreement with previous results [45, 46]. It is worth noting that the ycf1 gene was found at the JSA boundary from 1022 to 1034 bp in the IRA region in all cp genomes analyzed. Besides, the trnH gene located at the LSC region in all tested cp genomes, but the distance to the JLA boundary varies from 2-30bp. Combining the above results, we indicate that these seven cp genomes were relatively conserved, and the boundary divergence in the JSA and JLA in these species was the main reason for the expansion and contraction of the IR region.
